# Timing of Food Introduction and the Risk of Food Allergy

**DOI:** 10.3390/nu11051131

**Published:** 2019-05-21

**Authors:** Valentina Ferraro, Stefania Zanconato, Silvia Carraro

**Affiliations:** Women’s and Children’s Health Department, University of Padova, via Giustiniani 2, 35128 Padova, Italy; stefania.zanconato@aopd.veneto.it (S.Z.); silvia.carraro@unipd.it (S.C.)

**Keywords:** food allergy, complementary food, weaning, infants, cow milk protein, hen’s egg, peanuts, soy, wheat, fish

## Abstract

Given that the prevalence of pediatric IgE-mediated food allergies (FA) has followed a substantive increase in recent decades, nowadays, a research challenge is to establish whether the weaning strategy can have a role in FA prevention. In recent decades, several studies have demonstrated that delayed exposure to allergenic foods did not reduce the risk of FA, leading to the publication of recent guidelines which recommend against delaying the introduction of solid foods after 4–6 months of age, both in high- and low-risk infants, in order to prevent food allergy. In the present review, focusing on cow’s milk protein, hen’s eggs, peanuts, soy, wheat and fish, we describe the current scientific evidence on the relationship between timing of these foods’ introduction in infants’ diet and allergy development.

## 1. Introduction

The prevalence of Immunoglobulin E (IgE)-mediated food allergies (FA) in children showed a significant increase in the last decades [1]. Nowadays, in developed countries the estimates for the IgE-mediated FA in children is approximately 6–8%, with cow’s milk, hen’s eggs, soy, peanut, tree nuts, wheat, fish and seafood being the foods more often involved [1,2,3,4,5]. In the 1990s, food allergy prevention strategies were based on food avoidance in high-risk infants (i.e., with atopy in first-degree family members), and even in their mothers during pregnancy and breastfeeding [6,7,8]. The major scientific societies, as the American Academy of Pediatrics (AAP) [9], the American College of Asthma Allergy and Immunology [10] and the European Academy of Allergy and Clinical Immunology (EAACI) [11], used to recommend a late introduction of allergenic foods (e.g., milk after 12 months of age and egg after 24 months) on weaning for children at risk for allergy. These recommendations came from the assumption of a certain degree of “immaturity” of the mucosal immunity during infancy, which was supposed to allow an easier sensitization towards food antigens [12].

In 2008, a discussion paper explored the basis for concern over the recommendation for delayed complementary food introduction to prevent allergic disease, and suggested that tolerance to food might be regulated by an early and constant exposition to food proteins during a “critical window”, between 4 and 6 months of age [13]. In the meantime, several studies have been published demonstrating that delayed exposure to allergenic foods did not reduce the risk of FA [14,15,16,17,18,19,20,21]. In keeping with this evidence, in the latest recommendations (Table 1) issued by the World Health Organization (WHO) [22], the AAP [23,24], the EAACI [25], the European Society for Pediatric Gastroenterology, Hepatology and Nutrition (ESPGHAN) [26], and the European Food Safety Authority (EFSA) [27], it is reported that no scientific evidence justifies a delayed introduction of solid foods after 6 months of age to prevent allergy both in high and low-risk infants; at the same time, there are no scientific data supporting a role for early exposure -before 4 months of age- to main allergenic foods to prevent food allergy [28,29], as confirmed also by a recent metanalysis [30].

Here, we discuss presently available data on the relationship between timing of food introduction in infant diet and food allergy development.

## 2. Breast Milk and Cow’s Milk Protein 

### 2.1. Exclusive Breast-Feeding Regardless the Risk of FA

The natural first food for infants is breast milk and exclusive breast-feeding (EBF), as defined by the WHO, means that only breast milk is given to the infant and no other liquids or solids, except for vitamins, mineral supplements, or medicines [32]. EBF, by well-nourished mothers, is recommended for 6 months [32] (Table 1): during this time breast milk can provide all the energy and nutrients that the infant needs, such as protein, vitamins (except for vitamin K in the first weeks of life and vitamin D [33,34]) and minerals [26].

From a nutritional standpoint, a systematic review about the optimal duration of EBF, published in 2012 (23 eligible studies: 11 from low- and 12 from high-income countries, only 2 RCTs conducted in a low-income setting), compared EBF for 6 months versus EBF for 3 to 4 months followed by partial breast-feeding plus complementary food [35]. Authors demonstrated no risks in recommending exclusive breastfeeding for the first 6 months of life in both developing and developed-country settings [35]. In particular, the systematic review showed that there were no deficits in growth in both groups and infants who are exclusively breastfed for 6 months experienced less morbidity from gastrointestinal infection [35]. Nonetheless, the authors emphasize that infants should always be managed individually, to early identify possible inadequate growth or other adverse outcomes and offer appropriate measures in response to these problems [26,35]. Other authors underline that breast milk alone may not provide sufficient iron and zinc between the age of 4 and 6 months suggesting that there may be a beneficial effect from introducing complementary food alongside breast-feeding from 4 months of life, even in populations at low risk for iron deficiency [27,36,37,38,39,40].

From an allergologic standpoint, the current AAP and ESPGHAN recommendations suggest to continue breastfeeding while solids are introduced into the diet [23,41]. Likewise, a nested within a cohort case-control study found that children diagnosed with FA by 2 years of age were given solid food earlier (≤16 weeks) and were less likely to be receiving breast milk when cow’s milk protein was first introduced into their diet; in other words, continuing breastfeeding during solid food introduction and delaying such introduction until at least 17 weeks of age was associated with fewer FA [29]. However, a firm conclusion about the role of breast-feeding in preventing or delaying the onset of atopic diseases is not possible at this time, mainly because truly randomized breastfeeding goes beyond the limits of what is ethical and correct [23,24]. A recent systematic review reports that there is insufficient evidence to determine whether never vs. ever being fed human milk, or whether the duration of any human milk feeding, are associated with FA development [42].

### 2.2. Cow’s Milk Proteins Introduction Regardless the Risk of FA

When breastfeeding is not possible or not sufficient, the introduction of cow’s milk proteins occurs in the first days or weeks of life, through a cow’s milk formula, as recommended by the AAP [43]. Early exposure to cow’s milk proteins was closely studied, and, with respect to FA development, the results are inconsistent. A randomized trial [44] and a cohort study [45] suggested an increased risk of cow’s milk allergy if children were fed cow’s milk protein in the first few days of life. In contrast, a prospective noninterventional study demonstrated that infants regularly exposed to cow’s milk protein starting from the neonatal period rarely have IgE-mediated cow’s milk allergy, while they are at higher risk for this allergy if they are not regularly exposed to cow’s milk protein until the age of 4 to 6 months [46].

From a nutritional standpoint, as far as the whole cow’s milk is concerned, both AAP and ESPGHAN recommend that it should not be used as the main drink before 12 months of age [23,26,41], being poor in iron and possible cause of intestinal microhemorrhages [41,47]. Moreover, recent data suggest that consuming whole cow’s milk in infancy may stimulate rapid weight growth and possible overweight development [48].

### 2.3. High-Risk Infants

In infants at high risk of developing atopic disease (i.e., infants with at least one first-degree relative with documented allergic disease) there is evidence that EBF for at least 4 months decreases the cumulative incidence of atopic dermatitis and FA [23,49].

EAACI [25] recommend in high-risk infants (children with one first-degree relative with history of allergic disease) who cannot be breastfed, the use of hypoallergenic formula, and AAP [23] used to recommend in high-risk infants (i.e., infants with at least one fist-degree relative with documented allergic disease) extensively or partially hydrolyzed formula as a means to prevent the development of atopic diseases. However, the role for specific infant formulas to prevent allergy is now debated [50,51,52]. Indeed, a recent clinical report of the AAP written by Greer et al. states that nowadays there is no evidence on the role of partially or extensively hydrolyzed formula in the prevention of atopic disease in infants and children, even in those at high risk for allergic disease [24].

In keeping with this, a recent systematic review demonstrates that there is no significant difference in the risk of “any food allergy” using partially (risk ratio 1.73, 95% confidence interval 0.79 to 3.80; I2 = 42%) or extensively (0.86, 0.26 to 2.82; I2 = 42%) hydrolyzed formula compared to standard formula and no significant difference in the risk of allergic sensitization to cows’ milk with partially (1.30, 0.65 to 2.60; I2 = 0%) or extensively (0.77, 0.09 to 6.73; I2 = 77%) hydrolyzed formula [50]. In addition, a Cochrane published in 2018 concluded that, in infants unable to be exclusively breast fed, no substantial evidence supports prolonged feeding with a hydrolyzed formula instead of a cow’s milk formula for prevention of allergic disease, asthma, eczema, rhinitis or FA [53].

## 3. Hen’s Egg

In 2010, for the first time, a population-based, cross-sectional study suggested that the introduction of hen’s eggs to infant diets between 4 and 6 months might defend against egg allergy [54]. Lately, several trials have analyzed the relationship between timing of the introduction of eggs to the infant diet and the risk of allergic diseases (Table 2). Recently, a systematic review and meta-analysis analyzed these trials [55,56,57,58,59] (overall 1915 participants), concluding that they provide moderate-certainty evidence that early egg introduction—between 4 to 6 months—was associated with reduced egg allergy risk compared with later egg introduction (risk ratio [RR] 0.56 [95% CI 0.36–0.87], *P* = 0.009), with similar findings in studies undertaken in populations at normal-risk, high-risk, and very high-risk of allergy [60].

With regard to cooking, high rates of reaction are described on raw egg introduction [55,57] while cooked eggs are better tolerated [52,61].

## 4. Peanuts

Regarding the timing of peanut introduction into infant diet, two trials should be considered.

The first is a randomized, open-label, controlled trial (the Learning Early About Peanut allergy study, LEAP study) conducted by Du Toit et al. in 2015 [63] in “high-risk” infants aged 4 to 11 months with severe eczema, egg allergy, or both and with a skin-prick test for peanut allergy < 4 mm. Recruited children were randomly assigned to receive, up to the age of 5 years, 6 g of peanut protein per week (as peanut snack or peanut butter) or to avoid peanuts. The authors clearly showed that a sustained consumption of peanuts beginning in the first 11 months of life, was associated with a significant reduction of the proportion of children with peanut allergy at 5 years of age; in particular, in the subgroup with initial negative results on the skin-prick test, 13.7% of the children in the avoidance group and 1.9% of those in the consumption group (*P* < 0.001) were diagnosed with peanut allergy; in the group with initial wheal 1 to 4 mm on peanut skin-prick test, 35.3% of children in the avoidance group and 10.6% of those in the consumption group were diagnosed with peanut allergy (*P* = 0.004). The authors concluded that, in children at high-risk for allergy, the early introduction of peanuts significantly decreased peanut allergy [63]. Furthermore, authors pointed out that early consumption of peanut in high-risk infants is allergen specific and does not prevent the development of other allergic disease [64]. Later, a follow-up study published by the same authors in the same patients, demonstrated that in children at high risk for allergy in whom peanuts had been introduced in the first year of life and continued up to 5 years of age, a 12-month period of peanut avoidance was not associated with an increase in the prevalence of peanut allergy [65].

The second study is a randomized, controlled trial (the Enquiring About Tolerance, EAT) conducted by Perkin et al. in 2016 [58] in 3-month-old exclusively breastfed infants, who were randomly assigned to early introduction of six allergenic foods (peanut, cooked egg, cow’s milk, sesame, whitefish, and wheat) or to current practice (introduction at approximately 6 months of age). The intention to treat analysis, failed to show a protective effect against FA development for the early introduction of allergenic foods. Further analysis suggested that the possibility of preventing food allergy by means of the early introduction of multiple allergenic foods in normal breast-fed infants may depend on adherence and dose [58].

A recent metanalysis analyzed these two trials [58,63] giving the evidence that peanut introduction between 4 and 11 months of age was associated with lower risk of peanut allergy (RR, 0.29; 95% CI, 0.11–0.74; *P* = 0.009) [60]. The metanalysis authors, nonetheless, highlighted the high heterogeneity of these two trials, having that in the study by Du Toit et al. children showed a high treatment adherence compared with a more variable adherence in the study by Perkin et al. [58,60,63].

It is noteworthy that a consensus [66] published by 12 societies (i.e., American Academy of Pediatrics, American Academy of Allergy, Asthma & Immunology, American College of Allergy, Asthma & Immunology, Australasian Society of Clinical Immunology and Allergy, Canadian Society of Allergy and Clinical Immunology, European Academy of Allergy and Clinical Immunology, Israel Association of Allergy and Clinical Immunology, Japanese Society for Allergology, Society for Pediatric Dermatology, and World Allergy Organization) states that peanut introduction in “high-risk” (as defined by LEAP study) infant diet should be recommended between 4 and 11 months of age, keeping in mind that the whole peanut has to be avoided in children less than 4 years of age because of the risk of aspiration [66].

Based on existing data, the early introduction of peanut in high risk infants is current the measure of allergy prevention supported by the strongest evidence, and it has been proposed as a public health measure [52,67,68].

## 5. Soy

About the issue of timing for soy introduction in infants’ diet, no recent trials have been published.

A Cochrane published in 2006 suggested that feeding with soy formula should not be recommended for the prevention of atopy in infants at high risk (i.e, at least 1 first-degree relative with history of allergic disease or high cord IgE level) [69]. In keeping, a more recent randomized controlled trial, demonstrated that high risk infants (i.e, at least 1 first-degree relative with history of allergic disease) who had been given soy formula instead of conventional formula after stopping exclusive breast-feeding, were not at a lower risk of allergic manifestations (odds ratio, 1.26; 95% CI, 0.84–1.88) [70]. Eventually, both AAP and EAACI, state that in high-risk infants not breastfed, there is no convincing evidence to support the use of soy-based infant formula for allergy prevention [22,23].

It is known that soy formula can be used, if breastfeeding is not possible or not sufficient, in children with IgE-mediated cow’s milk allergy, as a second alternative (the first being a hydrolyzed infant formula) [71]. On the other hand, soy formula is not indicated in children with cow’s milk non-IgE-mediated allergic disorder, because up to 40% of infants react to both foods [72,73].

AAP and ESPGHAN recommend to postpone the introduction of soy-based formula in infants with IgE-mediated cow’s milk protein allergy after the first 6 months of life, based on the concern for an increased risk for soy allergy development [23,74] and for nutritional disadvantages (lower absorption of minerals and trace elements and high amounts of isoflavones) [74]. In contrast, a recent review did not support these recommendations, showing that evidence is lacking to demonstrate a higher risk of allergy when soy formula is used in infants younger than 6 months, even if the authors did not analyze possible nutritional disadvantages of soy-based formula [70].

## 6. Wheat and Fish

Concerning wheat and fish, Perkin et al. [58] analyzed, in a randomized controlled trial (the EAT study) conducted in 3-month-old exclusively breastfed infants, the effect of early introduction of these foods together with other four allergenic foods (peanut, cooked egg, cow’s milk, sesame), in comparison to the current practice in the United Kingdom (exclusive breast-feeding up to approximately 6 months of age; no consumption of fish or wheat before 5 months of age). In this trial, there were no cases of wheat allergy in either group, while the rate of fish allergy was non-significantly different in the early-introduction group and in the standard-introduction group (*P* = 1.00) [58].

Kull et al. [75] demonstrated in a prospective birth cohort study that early (during the first year of life) and regular fish consumption was associated with a reduced risk of allergic diseases (OR 0,76, 95% CI 0.61–0.94) and sensitization (OR 0,76, 95% CI 0.58–1) towards food and airborne allergens at 4 years of age. Another birth cohort study, published by Nwaru et al. [76], demonstrated that late introduction of solid foods was associated with increased risk of allergic sensitization to food and inhalant allergens; in particular, wheat introduction after 6 months of age was related to food allergen sensitization, whereas fish introduction after 8.2 months was related to aeroallergens sensitization [76].

Nonetheless, a recent metanalysis [60] concluded that, even if three cohort studies [75,76,77] found that early fish introduction (before the age of 6–9 months) was associated with reduced allergic sensitization to any allergen or food allergens, the evidence of the protective effect of such early introduction had very low certainty [60].

Further studies are needed to investigate pro and cons of early introduction of fish and wheat and to provide population-based recommendations for these foods [61].

## 7. Recommendations

**Figure 1:**
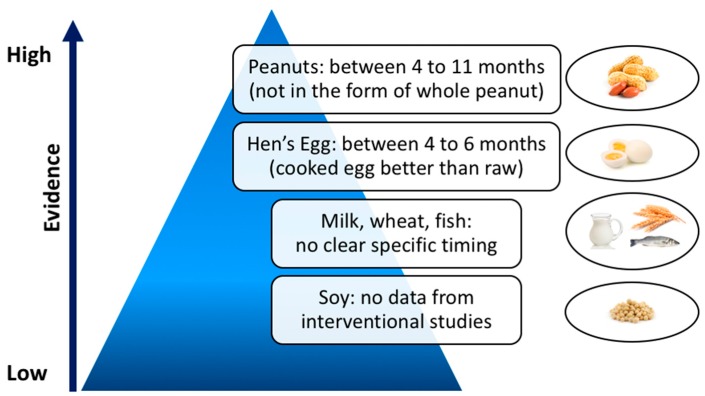
Timing of food introduction into infant diet and corresponding level of evidence.

Current international guidelines state that the introduction of allergenic foods does not need to be postponed beyond 4–6 months of age, both in high- and low-risk infants, but they provide no concrete guidance on whether these foods should be actively introduced within this time frame [23,24,25,26,27] (Table 1). Based on existing data, summarized in Figure 1, the only clear recommendation concerns early introduction of peanut between 4 and 11 months of age, as it is a proven prevention measure to reduce peanut allergy in infants at high risk [24,52,67,68]. Nonetheless, safety and practicality remain key issues, not completely clarified yet [78].

EBF, by well-nourished mothers, is recommended for 6 months [32,35], even if from 4 months of life there may be some beneficial effect of introducing complementary food, especially in order to provide the right demand of iron and zinc [27,36,37,38,39,40]. Solid foods should not be introduced before 4 to 6 months of age and breastfeeding should be continued while solids are introduced into the diet [23,41,79].

The introduction of cow’s milk proteins is recommended if breastfeeding is not possible or not sufficient, given that cow’s milk formula is the type of formula recommended by the AAP [43]. In high-risk infants not breastfed, there is no evidence on the role of partially or extensively hydrolyzed formula in the prevention of atopic diseases [24,50,53,80]. As far as the whole cow milk is concerned, both AAP and ESPGHAN recommend not to use whole cow milk as the main drink before 12 months of age [23,26,41].

Regarding the introduction of eggs, there is moderate-certainty evidence that early egg introduction at 4 to 6 months is associated with reduced egg allergy risk, with similar findings in studies undertaken in populations at normal-risk, high-risk, and very high-risk of allergy [60].

## 8. Conclusions

The available studies suggest that the introduction of allergenic foods should not to be postponed beyond 4–6 months of age, both in high- and low-risk infants. Nonetheless, based on existing data, the only clear recommendation concerns the early introduction of peanut between 4 and 11 months of age as a prevention measure to reduce food allergies in infants at high risk, even if the safety and practicality of doing so has not yet been fully explored.

## Figures and Tables

**Table 1:** Timing of exclusive breastfeeding and complementary food introduction.

	Exclusive Breastfeeding	Complementary Food
World Health Organization (WHO) [22]	For the first 6 months of life	All infants should start receiving foods in addition to breast milk from 6 months onwards
American Academy of Pediatrics (AAP) [23,24,31]	Exclusive breastfeeding for about 6 months, with continuation of breastfeeding for 1 year or longer as mutually desired by mother and infant	Although solid foods should not be introduced before 4 to 6 months of age, there is no current convincing evidence that delaying their introduction beyond this period has a significant protective effect on the development of atopic disease
European Academy of Allergy and Clinical Immunology (EAACI) [25]	Exclusive breastfeeding is recommended for the first 4–6 months of life	Introduction of complementary foods after the age of 4 months for all children irrespective of atopic heredity
European Society for Pediatric Gastroenterology, Hepatology, and Nutrition (ESPGHAN) [26]	Exclusive or full breast-feeding should be promoted for at least 4 months (17 weeks, beginning of the 5th month of life). Exclusive or predominant breast-feeding for approximately 6 months is considered a desirable goal.	Complementary foods should not be introduced before 4 months but should not be delayed beyond 6 months
European Food Safety Authority (EFSA) [27]	Exclusive breast-feeding is nutritionally adequate up to 6 months for the majority of infants, while some infants may need complementary foods before 6 months (but not before the age of 4 months) in addition to breastfeeding to support optimal growth and development	The introduction of complementary food into the diet of healthy term infants between the age of 4 and 6 months is safe and does not pose a risk for adverse health effects

**Table 2:** Characteristics of Randomized Clinical Trials of Egg Introduction and Risk of Egg Allergy.

Author, Year, Trial Name	Country	Population	Intervention	Outcome	Results
Bellach 2017, Hen’s Egg Allergy Prevention (HEAP) [55]	Germany (Berlin)	“Normal-risk” infants aged 4–6 months with specific IgE to egg < 0.35 kU/L	Pasteurized egg white powder (2.5 g protein) vs. rice powder 3 times/week from age 4–6 months to 12 months	Egg allergy diagnosed by oral food challenge at 1 year plus specific IgE to egg ≥ 0.35 kU/L	In egg group, 2.1% were confirmed to have egg allergies versus 0.6% in the placebo group (relative risk, 3.30; 95% CI, 0.35–31.32; *P* = 0.35)
Natsume 2017, Prevention of egg allergy with tiny amount intake trial (PETIT) [56]	Japan (Tokyo)	“High-risk” infants aged 4–5 months of age with atopic dermatitis	Heated egg powder, 50 mg/day, from 6–9 months; 250 mg/day from 9–12 months vs. placebo from 6–12 months	Egg allergy diagnosed by oral food challenge at 1 year	In the egg group 8% had an egg allergy compared with 38% in the placebo group (risk ratio 0.221; 95% CI, 0.090–0.543; *p* = 0.0001)
Palmer 2013, Solid Timing for Allergy Research (STAR) [57]	Australia (University of Western Australia)	“High-risk” singleton term infants with moderate or severe eczema (SCORAD ≥ 15) and no prior egg or solid food intake	One teaspoon pasteurized whole egg powder daily (0.9 g protein) vs. rice flour powder from age 4 months to 8 months	Egg allergy diagnosed by oral food challenge to pasteurized egg at 1 year plus positive skin prick test	In the egg group 33% were given a diagnosis of IgE-mediated egg allergy compared with 51% in the control group (relative risk, 0.65; 95% CI, 0.38–1.11; *P* = 0.11).
Palmer 2017, Starting Time for Egg Protein (STEP) [62]	Australia (University of Western Australia)	“High-risk” infants with an atopic mother, no prior egg ingestion, and no prior allergic disease	Pasteurized whole egg powder daily (0.9 g protein) vs. rice powder daily from age 4–6 mo to 10 mo	Egg allergy diagnosed by oral food challenge to pasteurized egg at 1 year plus positive skin prick test	In the egg group 7% were given a diagnosis of IgE-mediated egg allergy compared with 10.3% in the control group (adjusted relative risk, 0.75; 95% CI, 0.48–1.17; *P* = 0.20)
Perkin 2016, Enquiring about tolerance (EAT) [58]	United Kingdom (London)	“Normal-risk” singleton term infants exclusively breastfed for ≥3 months	Sequential introduction of 6 allergenic foods (4 g protein/week for each food, yogurt, peanut, boiled egg, sesame, fish, and wheat) from age 3 months, vs. avoidance to age ≥ 6 months	Egg allergy diagnosed by oral food challenge to egg at 1 and at 3 years	- intention-to-treat analysis: egg allergy 3.7% in the early-introduction group and 5.4% in the standard-introduction group, i.e., a nonsignificant 31% lower relative risk in the early-introduction group (*P* = 0.17) - In the per-protocol analysis: egg allergy 1.4% in the early-introduction group versus 5.5% in the standard-introduction group, representing a 75% lower relative risk (*P* = 0.009)
Tan 2017, Beating Egg Allergy (BEAT) [59]	Australia (Sydney)	“High-risk” infants with first-degree relative with allergic disease and egg skin prick test < 2mm at age 4mo	Pasteurized whole egg powder daily (350 mg egg protein) vs. rice powder daily from the time of solid food introduction to age 8 months	Egg allergy diagnosed by oral food challenge to lightly cooked whole egg at 1 year	Sensitization to egg white at 12 months was 20% and 11% in infants randomized to placebo and egg, respectively (odds ratio, 0.46; 95% CI, 0.22–0.95; *P* = 0.03)
